# The Molecular Basis of Sarcoma

**DOI:** 10.1155/2011/864130

**Published:** 2011-06-07

**Authors:** Stephen L. Lessnick, Heinrich Kovar, Peter Houghton

**Affiliations:** ^1^Huntsman Cancer Institute, Center for Children's Cancer Research, 2000 Circle of Hope, Salt Lake City, UT 84112, USA; ^2^Children's Cancer Research Institute, St. Anna Kinderkrebsforschung, 1090 Vienna, Austria; ^3^Center for Childhood Cancer, Nationwide Children's Hospital, 700 Children's Drive, Columbus, OH 43205, USA

The sarcoma field is at a crossroads. There have been incredible advances in the identification and characterization of key genetic events associated with sarcoma development. For example, many chromosomal translocation breakpoints have been cloned, and the fusion proteins associated with those breakpoints have been subjected to rigorous molecular analysis. Similarly, there has been an explosion of molecularly targeted agents available for the treatment of patients with cancer. In cases where these targeted agents inhibit a key abnormality in sarcoma, such as activated KIT in gastrointestinal stromal tumor, they have been quite successful. Unfortunately, examples of such success are still limited. Many investigators in the field have hoped that deeper understanding of the molecular basis of sarcoma may lead to new therapeutic opportunities for this varied set of diseases.

It is with this spirit that the current special issue is presented. Twenty-three papers are included, which cover a vast range in the field and include a mix of both original research and timely reviews. Through the process of presenting a broad array of molecular topics related to sarcoma development, it is our hope that investigators and other interested parties will recognize both common threads and unique issues across many different subtypes of sarcoma and that such recognition will stimulate new research directions that will lead to new cures for patients suffering from these diseases.

In the first paper of the special issue, “*Epigenetic regulation of apoptosis and cell cycle in osteosarcoma*,” Kleinerman and K. Rao-Bindal critically discuss frequency and prognostic impact of epigenetic inactivation of p16/p14ARF, HIC1, and RASSF1A in this disease, as well as the possible role of histone H3 lysine 27 monomethylation in osteosarcoma apoptosis. This provides unique insights into the role of epigenetics as a molecular basis for osteosarcoma development.

High-dose methotrexate is a mainstay of modern osteosarcoma therapy. While osteosarcomas respond well to high-dose methotrexate, they do not respond well to conventional doses of the drug. The second article, “*Impairment of methotrexate transport is common in osteosarcoma tumor samples*” by R. Sowers et al., demonstrates that methotrexate transport is impaired in high-grade osteosarcoma. This suggests that high doses of the agent are required to overcome this transport impairment, and implies that antifolate agents that are not dependent on the common transport pathway might be more effective for this disease.

In the third article, “*The molecular pathogenesis of osteosarcoma: a review*,” M. L. Broadhead et al., provide an extensive and timely discussion of the current knowledge on the etiology of the disease and more frontline translational studies aiming at targeted osteosarcoma therapy.

In the next review, “*Osteosarcomagenesis: modeling cancer initiation in the mouse*,” K. B. Jones traces the historical pathways for developing nonclinical models of osteosarcoma to the more contemporary genetically engineered mouse models and how they recapitulate the human disease. The work presents early approaches to creating non-clinical models that involved random mutagenesis, bone seeking radionuclides, external beam radiation, viral insertional mutagenesis and how these models informed human osteosarcoma. The review also details current approaches using gene targeting to engineer mouse models, and their value and limitations.

Although osteosarcoma displays features of poorly differentiated osteoprogenitors, the exact histogenesis is still unknown. In the fifth article, “*Defective osteogenic differentiation in the development of osteosarcoma*,” W. R. Wagner et al., assess the problem of osteosarcoma pathogenesis from a developmental angle. In their review they discuss osteosarcoma as a disease of impaired differentiation, and summarize the rationale and first data on the use of differentiation inducing agents as a potential therapeutic strategy in this disease. 

Understanding normal development and how these processes go awry in cancer has important implications for understanding tumorigenesis, and for considering new therapeutic approaches. With this in mind, the sixth article, “*The role of RUNX2 in osteosarcoma oncogenesis*,” by J. W. Martin et al., reviews the RUNX2 transcription factor and its potential role in osteosarcoma. RUNX2 is a DNA-binding transcription factor that is involved in normal bone development. RUNX2 is often overexpressed in osteosarcoma, and so its normal functions may also be important for the development of the tumor.

Continuing on with the osteosarcoma theme, the seventh article, “*Using epidemiology and genomics to understand osteosarcoma etiology*,” by S. A. Savage and L. Mirabello, provides a comprehensive review of the epidemiology of osteosarcoma, and also reviews the known genomic mutations and variants that are associated with osteosarcoma development. This review is particularly timely given expanding interest in large-scale studies of pediatric cancer epidemiology and genomics.

Transcription factor fusions involving the EWS gene or one of its relatives FUS or TAF15 in sarcomas or rare leukemias are considered dominant oncogenes. However, since EWS family proteins are ubiquitously expressed housekeeping proteins, allelic rearrangements may result in haploinsufficiency or even a dominant negative effect on the remaining expressed allele. Nuclear localization is considered an essential prerequisite for the normal function of EWS family proteins. In the eighth paper, “*Tyrosine phosphorylation in the C-terminal nuclear localization and retention signal (C-NLS) of the EWS protein*,” R. P. Leemann-Zakaryan et al., describe the role of C-terminal phosphorylation in physiological control of EWS nuclear localization, which is lost upon rearrangement with a transcription factor moiety in sarcoma.

In the ninth article, “*Dr. Jekyll and Mr. Hyde, the two faces of theFUS/EWS/TAF15 protein family*,” H. Kovar reviews the interesting dual personality of the FUS/EWS/TAF15 proteins. On the one hand, these proteins have a variety of wild-type functions that are important to normal cellular behavior that are slowly being worked out. On the other hand, they are evil fusion partners in oncogenesis. It seems likely that the oncogenic functions (both transcriptional and nontranscriptional) are related to the normal functions of these proteins.

In the next article, “*Copy number alterations (CNAs) and methylation in Ewing's sarcoma*,” M. S. Jahromi et al., provide a comprehensive literature review of available data reporting copy number alterations (CNA's), alterations in mitochondrial DNA, and gene silencing by methylation for Ewing's sarcoma. The potential implication of trisomy 8 and 12, gains on chromosome 2q, and deletion or methylation silencing of the CDKN2A (p16-INK4a) locus, or genes involved in the extrinsic death pathway for patient outcome are reviewed. This work provides an overview of intriguing data, limited by sample size, that points to the pathogenesis of Ewing's sarcoma, and the potential to impact treatment outcome through large-scale studies that are now possible using archival tissue.

In the eleventh article, “*Targeting angiogenesis in childhood sarcomas*,” H. K. Bid and P. J. Houghton provide a timely and comprehensive evaluation of the literature regarding angiogenesis and vasculogenesis in sarcomas (particularly, pediatric sarcomas). In addition to reviewing the data regarding the biologic basis for these processes, the authors also review a series of therapeutic strategies based on inhibiting angiogenesis and vasculogenesis, in sarcoma.

In the twelfth article, “*Immune-based therapies for sarcoma*,” S. M. Pollack et al., point out that there are great unmet needs in the systemic therapy of sarcomas and that nonchemotherapeutic strategies might be exploited for this role. The authors provide an important review of immunotherapy in sarcoma and discuss a variety of therapeutic trials and concepts in the field, including non-specific immunomodulation and targeted immunotherapy approaches. This review highlights the opportunities, and remaining challenges, that exist in allowing immunotherapy to become a part of the armamentarium to treat sarcomas in the future.

IGF signaling is an important component of the machinery driving cellular growth in embryonal tissues and in many tumors. It was therefore expected that anti-IGF therapy should be a promising therapeutic option in the treatment of cancer, particularly of sarcomas in which IGF signaling is constitutively activated. In the thirteenth article, “*Targeting the insulin-like growth factor pathway in rhabdomyosarcomas: rationale and future perspectives*,” A. S. Martins et al., discuss the role of IGF signaling in rhabdomyosarcoma and summarize first clinical experience and so far unexplored options of combination chemotherapy.

The article by L. E. S. Crose and C. M. Linardic, “*Receptor tyrosine kinases as therapeutic targets in rhabdomyosarcoma*,” reviews the current knowledge regarding expression of tyrosine kinase receptors in rhabdomyosarcoma. The review presents up-to-date information on expression in clinical samples of specific receptor kinases including members of the epidermal, hepatocyte, fibroblast, platelet and insulin-like growth factors and their potential role in tumorigenesis as shown in genetically engineered models. The review details current ongoing clinical studies of agents that target these receptors and discusses the future development of these agents in the context of contemporary therapeutic approaches to rhabdomyosarcoma. 

Among the various cellular stresses which tumor cells have to evade in order to survive and proliferate is a markedly increased production of reactive oxygen species (ROS). In the fifteenth paper, “*The role of mirk kinase in sarcomas*”, E. Friedman discusses how the activity of the serine/threonine kinase Mirk/dyrkB may prevent apoptosis of osteosarcoma and rhabdomyosarcoma cells by increasing the expression of antioxidant scavenger proteins. Dr. Friedman reviews the literature on the role of Mirk as a potential prognostic biomarker in osteosarcoma, and on the therapeutic promise of Mirk inhibition in combination of chemotherapy with conventional anticancer drugs.

The sixteenth paper, “*miRNA profiling: how to bypass the current difficulties in the diagnosis and treatment of sarcomas*,” A. Gougelet et al., demonstrate that real-time quantitative PCR approaches focused on microRNA (miRNA) signatures provide a new prognostic and diagnostic approach in two important sarcoma types: osteosarcoma and rhabdomyosarcoma. In the case of osteosarcoma, miRNA profiles changed in unique and predictive ways following exposure to chemotherapeutic agents, which suggests that such profiles might be used in a prognostic fashion. In the case of rhabdomyosarcoma, miRNA profiles were diagnostic of each of the subtypes of rhabdomyosarcoma. Thus, miRNA profiling may have important use in the diagnostic and prognostic analysis of at least some types of sarcoma.

The next paper, “*Delineation of chondroid lipoma; an immunohistochemical and molecular biological analysis*,” by de Vreeze et al., presents a study from the Dutch Pathology Registry of chondroid lipomas, extremely rare benign tumors. Chondroid lipoma may exhibit histologic features resembling myoepithelioma, myxoid liposarcoma, extraskeletal myxoid chondrosarcoma, hibernoma, and other lipomatous or chondroid neoplasms, resulting in difficulties in accurate diagnosis with an appropriate treatment. The aim of this study was to delineate chondroid lipoma from several morphologic mimics by the means of immunohistochemistry. Although these tumors show high expression of CCND1, the authors rule out the CCND1 and FUS genes as candidates involved in the t(11;16)(q13;p13) previously reported as a recurrent translocation in this rare benign lipomatous tumor.

While chondrocytes are mesenchymal in nature, chondrosarcomas exhibit features of epithelial cells as well. The eighteenth article, “*Human chondrosarcoma cells acquire an epithelial-like gene expression pattern via an epigenetic switch: evidence for mesenchymal-epithelial transition during sarcomagenesis*,” by M. P. Fitzgerald et al., provides evidence for epigenetic activation of a set of epithelial markers in chondrosarcomas, and downregulation of snail, as compared to chondrocytes. These data suggest that chondrosarcomas undergo a mesenchymal to epithelial transition via an epigenetic pathway.

In the next article,* “Spinal chondrosarcoma: a review,” *P. Katonis and colleagues present a comprehensive review of spinal chondrosarcoma, a rare variant comprising 10 percent of chondrosarcoma patients. The review covers histologic classification and molecular characteristics associated with disease progression, subtype classification, and risk factors. Radiologic features associated with diagnosis and staging and current approaches to therapy and prognosis are discussed.

Dermatofibrosarcoma protuberans (DFSP) is a rare cutaneous-origin sarcoma associated with constitutive activation of the receptor tyrosine kinase (RTK) PDGFR by chromosomal rearrangement with the collagen gene COL1A1. In the twentieth paper, “*Advances in molecular characterization and targeted therapy in dermatofibrosarcoma protuberans (DFSP)*,” P. Rutkowski et al., discuss their clinical results obtained with the broad spectrum RTK inhibitor Imatinib in the treatment of inoperable and/or metastatic and/or recurrent cases of DFSP.

Clinically, distinguishing benign uterine leiomyoma from malignant uterine leiomyosarcoma (LMS) remains a challenge. The next article, “*Molecular approach to uterine leiomyosarcoma: LMP2-deficient mice as an animal model of spontaneous uterine leiomyosarcoma*,” by T. Hayashi et al., reviews the molecular pathogenesis of LMS, with specific reference to the role of LMP2, a gene encoding a component of the immunoproteasome, in development of uterine leiomyosarcoma. They report that uterine LMS occurred in female LMP2-deficient mice at age of 6 months and the incidence at 14 months of age was about 40%. They identify LMP2, a single IFN-*γ*-responsive gene product, as obligatory for tumor surveillance and demonstrate a tissue-specific role for LMP2 in protection from spontaneous neoplasms of the uterus. The potential for use of LMP2 expression as a diagnostic marker to distinguish leiomyoma from LMS is proposed.

Recently, great advances have been made in understanding the molecular basis of different types of liposarcoma. In the twenty-second article, “*Liposarcoma: molecular genetics and therapeutics*,” S. Young et al., review the molecular basis for liposarcoma, with a focus on recent molecular genetic data from techniques such as fluorescent in situ hybridization (FISH) and comparative genomic hybridization (CGH). In some cases, these molecular changes (such as amplification of MDM2 and CDK4) suggest rational therapeutic approaches for these diseases, which are also reviewed.

Sarcomas tend to metastasize to lungs and bones, and prevention of metastasis is considered the holy grail of cancer treatment. In the final article of the issue, “*The role of chemokine receptor CXCR4 in the biologic behavior of human soft tissue sarcoma*”, R. H. Kim et al., review the growing body of evidence that chemokine receptors, specifically CXCR4, play an important role in homing of sarcoma cells to lung and bones. Data are discussed that imply CXCR4 inhibitors as promising add-ons to classical chemotherapy to prevent deadly metastases in sarcoma patients. 

Clearly there are great challenges, but also great opportunities, to link the molecular basis of sarcoma (and all of its relevant associated phenotypes) to new diagnostic, prognostic, and therapeutic approaches for this complex group of malignancies. We hope that the articles in this special issue provide a strong stimulus for such a linkage and will help to spur ongoing advances that will ultimately transform the care of patients with sarcoma.

*Stephen L. Lessnick* *Stephen L. Lessnick* *Heinrich Kovar* *Heinrich Kovar* *Peter Houghton* *Peter Houghton*

